# Supplementary material for: Structural and functional analysis of hyper-thermostable ancestral L-amino acid oxidase that can convert Trp derivatives to D-forms by chemoenzymatic reaction
Source: Commun Chem. 2023 Sep 22;6:200. doi: 10.1038/s42004-023-01005-1 (PMC10517122; doi:10.1038/s42004-023-01005-1)
Supplement: Supplementary file 2 — Supplementary Information [file 42004_2023_1005_MOESM2_ESM.pdf]

Supporting Information

Structural and functional analysis of hyper-thermostable ancestral L-amino acid oxidase that can convert Trp derivatives to D-forms by chemoenzymatic reaction

Yui Kawamura[1, 4], Chiharu Ishida[1,4], Ryo Miyata[1#], Azusa Miyata[1], Seiichiro Hayashi[2], Daisuke Fujinami[1], Sohei Ito[1] and Shogo Nakano[1,3*]

[1]Graduate Division of Nutritional and Environmental Sciences, University of Shizuoka, 52-1 Yada, Suruga-ku, Shizuoka 422-8526, Japan

[2]Division of Structural Biology, Medical Institute of Bioregulation, Kyushu University, Higashi-ku, Fukuoka 812-8582, Japan

[3]PREST, Japan Science and Technology Agency, Saitama, Japan

[4]These authors contributed equally to this work

[#]Current address: Health and Medical Research Institute, National Institute of Advanced Industrial Science and Technology, 2217-14 Hayashi-cho, Takamatsu, Kagawa 761-0395, JAPAN

[*]Correspondence to Shogo Nakano (snakano@u-shizuoka-ken.ac.jp)

Keywords: L-amino acid oxidase, crystal structure, ancestral sequence reconstruction, deracemization

Contents

・**Supporting Figures** **S3-S7**

Schematic views indicating how to design HTAncLAAO2 S3

Gel-filtration chromatography of HTAncLAAO2(native), K304A and C505A variants S4

Active site structures of four distinct L-amino acid oxidases S5

Enzyme kinetic plots of HTAncLAAO2 variants (L320A and Y383A) S6

Chemical characterization of D-1a to 1c S7

・**Table contents** **S8-S15**

Four homologs sequences to design HTAncLAAO2 by ASR S8

Protein and DNA sequence of HTAncLAAO2 S9

Summary of soluble expression level of HTAncLAAO2 and other four LAAOs S10

X-ray crystallographic parameters of HTAncLAAO2 S11

Relative activity of HTAncLAAO2 and the variants toward 20 L-amino acids S12

Enzyme kinetic parameters of HTAncLAAO2 and their variants S13

Liquid chromatography condition and retention time for D,L-**1a**-**1c** S14

Primer list to design HTAncLAAO2 variants S15

・**Supplementary references** **S16**

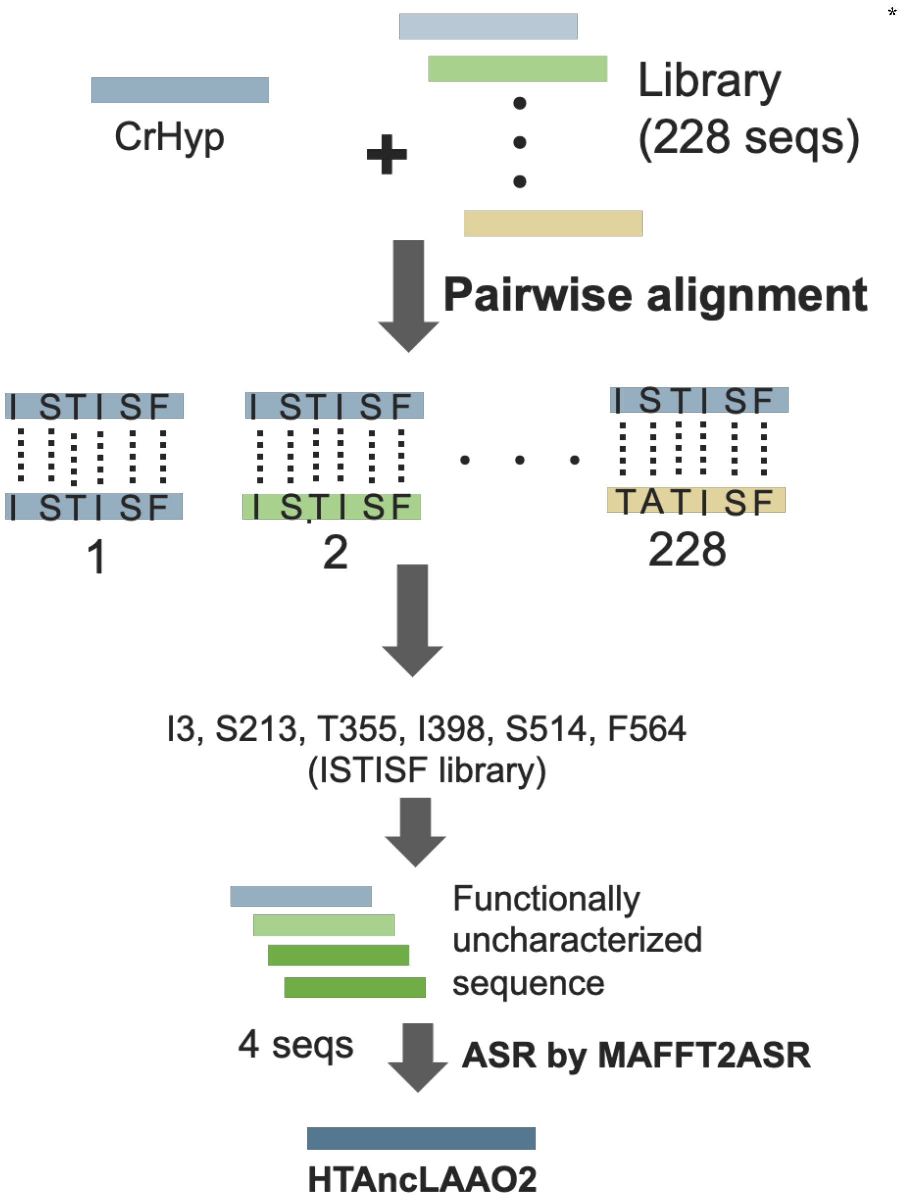

Fig. S1. Schematic view for designing how to design HTAncLAAO2. Hypothetical protein from Caulobacter radius (CrHyp, PMID: WP_116490310.1) were utilized as a template. Based on the six residues (I3, S213, T355, I398, S514 and F564) as a motif, we selected total four sequences (Table S1). ASR was adopted to the four sequences, and finally, we obtained protein sequence of HTAncLAAO2 (Table S2).

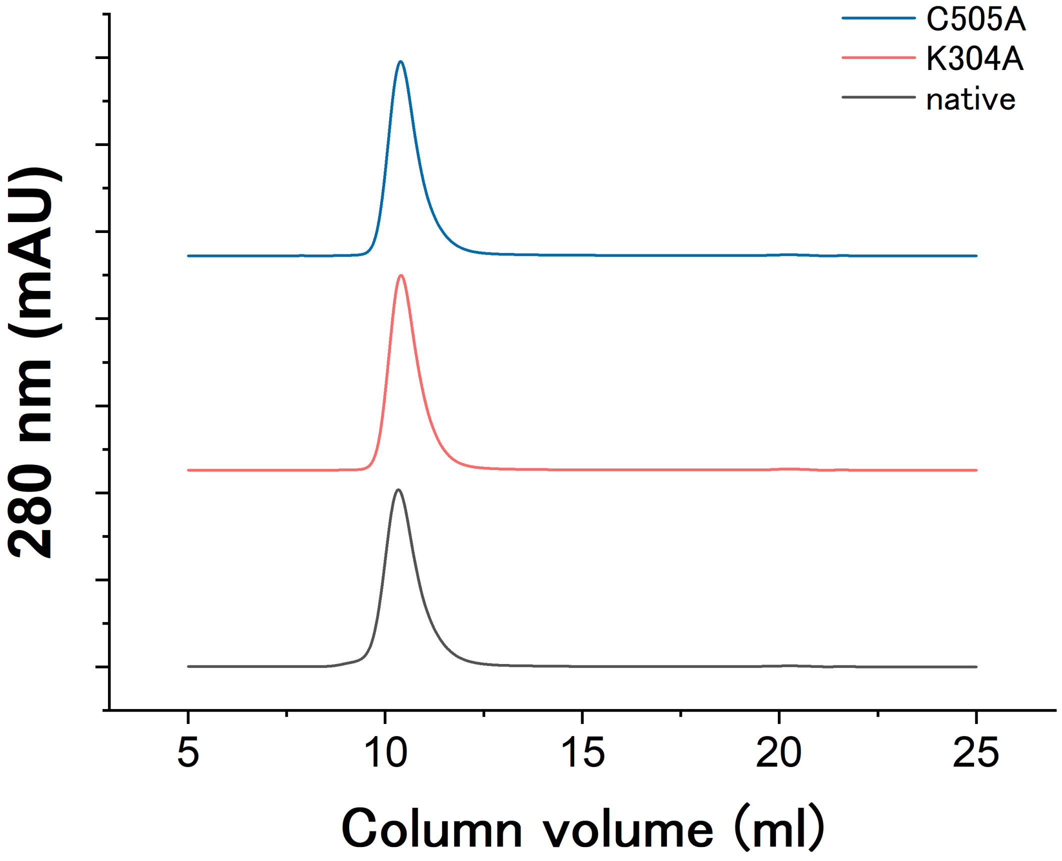

Fig. S2. Gel-filtration chromatography of HTAncLAAO2(native), K304A and C505A variants. As shown in the figure, the elution volume of the HTAncLAAO2(native) (10.34 mL, black line), K304A (10.39 mL, red line) and C505A (10.40 mL, blue line) was almost identical to each other, suggesting that the NOS bridge does not affect to the formation of oligomeric state.

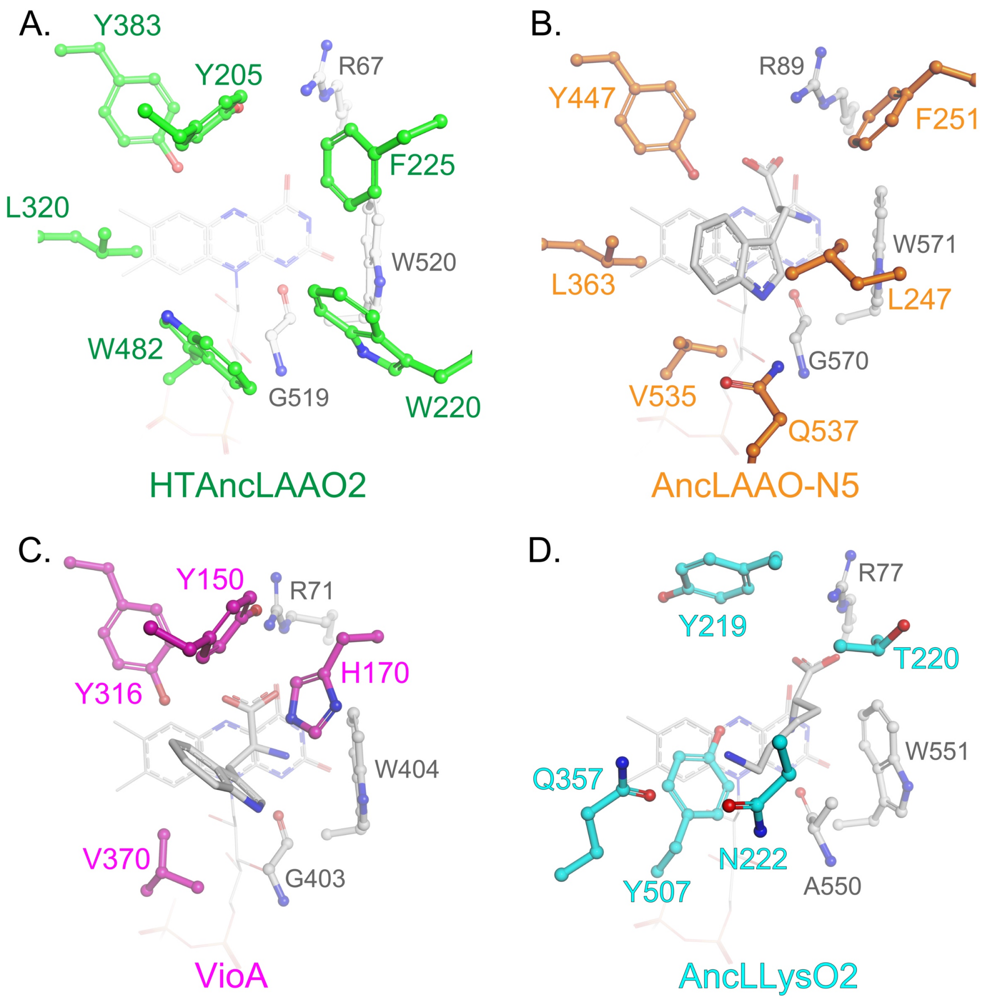

Fig. S3, Active site structures of HTAncLAAO2 (A), AncLAAO-N5 (B, PDB ID: 7C4M), VioA (C, PDB ID: 5ZBD), and AncLLysO2 (D, PDB ID: 7X7J). HTAncLAAO2 and AncLAAO2-N5 exhibited broad substrate selectivity toward various L-amino acids, whereas VioA and AncLLysO2 had the high specificity toward L-Trp and L-Lys, respectively.

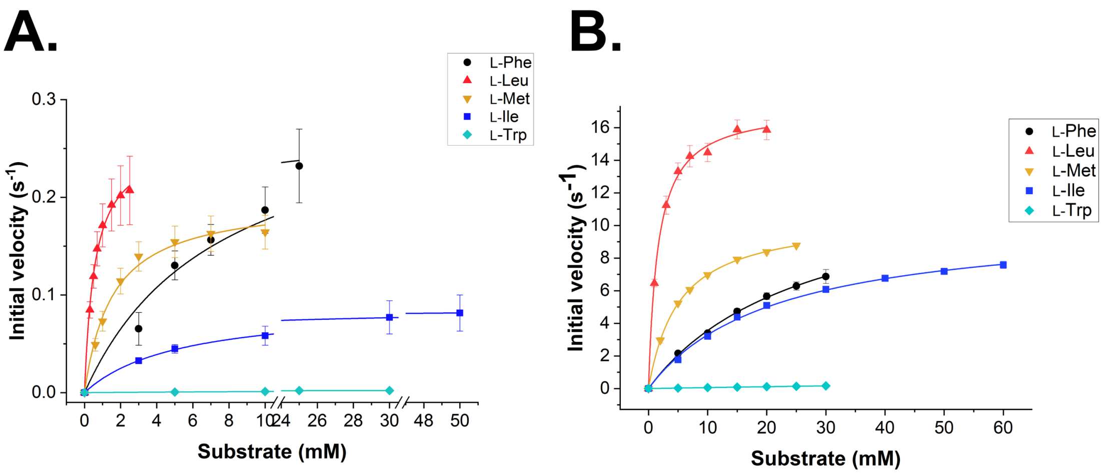

Fig. S4. Enzyme kinetics plots of L320A (A) and Y383A (B) variants of HTAncLAAO2. Initial velocities, which are obtained utilizing L-Phe, L-Leu, L-Met, L-Ile and L-Trp as substrates, are plotted as black circle, red triangle, orange downward triangle, blue square and light blue diamond, respectively. All measurements were performed independently three times (N = 3).

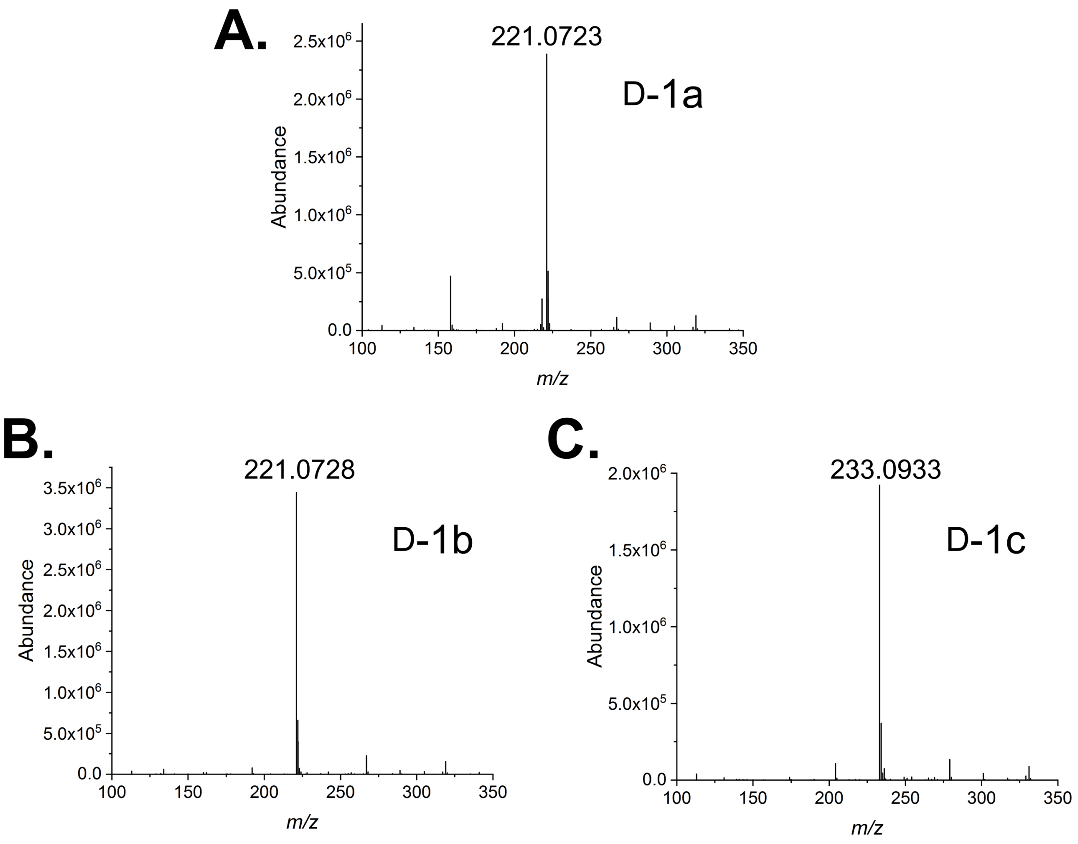

Fig. S5. HRMS spectra of D-1a (A), D-1b (B) and D-1c (C). Extracted ion chromatogram of $[M+H]^-$ is indicated in the figure.

Table S1. Four homolog sequences that were utilized to design highly thermostable ancestral L-amino acid oxidase 2 (HTAncLAAO2)

Accession	Locus tag	Species
WP_129538518.1	FAD-dependent oxidoreductase	*Flavobacterium sp. 140616W15*
WP_052250020.1	FAD-dependent oxidoreductase	*unclassified Flavobacterium*
WP_035627035.1	FAD-dependent oxidoreductase	*Flavobacterium hydatis*
WP_143379052.1	FAD-dependent oxidoreductase	*Flavobacterium sp. ZT3R18*

Table S2. Protein and DNA sequences of HTAncLAAO2

>HTAncLAAO2
MENEKIDIAIVGGGVSGVYSAWKLKTKYPNKKIVLFEGGDHIGGRLLSVIPPGIPNMVAEL
GGMRILENTQKLIVKLIDDINEKLSQEDQIELYDFPVDQPQNIAYLRGEHLRLFDFTNDPD
KVPYKLSFLEKGNTSGTIIVNAIEQLVPGITNTDLTEEERLKMCQEATFEGAPLYTLGFWN
LLYRVISGEAYQFSIDSGGYNSTLVNWNAADAIPWYLSDFGIKPVYKGFKNGFQQVPISL
ANFFEEDGGEIRLNAKLEGFEFKNNLFELTIDGEIIEATQLILAMPRRSLDLLTNTSPKLQEI
QSLIGSVTPRPLFKVFTTYSSPWWRNAGYTDSEGGYIPLQSGRTVTDLPIRQTYYWPKN
NGQPSVSGESMLLASYDDGSNIGFWDGLRPQRKKAWKKGLSHAELADDPFIGEYSETE
SLLSKALNQTWHQYKAPRKMVEELSRQLKQIHDVDYTPAVKNASFRDWGEDPFGGGW
NSWNIGVKSWEVKEKIVHPIDNCSLYICGEAYSDGQGWVEGALQTADIMLKKFIAVESKT
SVKEEAILE

>HTAncLAAO2-DNA
ATGGGCATGGAAAACGAGAAAATCGATATTGCGATCGTTGGTGGCGGTGTGAGCGG
CGTTTACAGCGCGTGGAAGCTGAAAACCAAGTATCCGAACAAGAAAATTGTGCTGTT
CGAAGGCGGTGACCACATCGGCGGTCGTCTGCTGAGCGTTATTCCGCCGGGTATCC
CGAACATGGTGGCGGAACTGGGCGGTATGCGTATTCTGGAGAACACCCAGAAACTG
ATCGTTAAGCTGATTGACGATATCAACGAAAAGCTGAGCCAGGAAGATCAAATTGAG
CTGTACGACTTCCCGGTGGATCAGCCGCAAAACATCGCGTATCTGCGTGGTGAGCA
CCTGCGTCTGTTCGACTTTACCAACGACCCGGATAAAGTTCCGTACAAGCTGAGCTT
TCTGGAAAAGGGTAACACCAGCGGCACCATCATTGTTAACGCGATTGAGCAGCTGG
TGCCGGGTATCACCAACACCGATCTGACCGAGGAAGAGCGTCTGAAGATGTGCCAA
GAAGCGACCTTTGAGGGTGCGCCGCTGTACACCCTGGGTTTTTGGAACCTGCTGTA
CCGTGTTATTAGCGGCGAGGCGTATCAGTTCAGCATCGACAGCGGCGGTTATAACAG
CACCCTGGTGAACTGGAACGCGGCGGACGCGATTCCGTGGTACCTGAGCGATTTTG
GTATCAAACCGGTTTATAAAGGTTTCAAGAACGGCTTTCAGCAAGTGCCGATTAGCCT
GGCGAACTTCTTTGAAGAGGATGGCGGTGAAATCCGTCTGAACGCGAAACTGGAAG
GCTTCGAGTTTAAGAACAACCTGTTTGAGCTGACCATTGATGGTGAAATCATTGAAG
CGACCCAGCTGATCCTGGCGATGCCGCGTCGTAGCCTGGACCTGCTGACCAACAC
CAGCCCGAAACTGCAGGAAATTCAAAGCCTGATCGGTAGCGTTACCCCGCGTCCGC
TGTTCAAGGTGTTTACCACCTACAGCAGCCCGTGGTGGCGTAACGCGGGTTACACC
GATAGCGAGGGCGGTTATATTCCGCTGCAGAGCGGTCGTACCGTTACCGACCTGCC
GATCCGTCAGACCTACTATTGGCCGAAGAACAACGGCCAACCGAGCGTGAGCGGTG
AAAGCATGCTGCTGGCGAGCTATGACGATGGTAGCAACATTGGCTTCTGGGACGGT
CTGCGTCCGCAGCGTAAGAAAGCGTGGAAGAAAGGCCTGAGCCACGCGGAACTGG
CGGACGATCCGTTTATCGGCGAGTACAGCGAAACCGAGAGCCTGCTGAGCAAAGCG
CTGAACCAGACCTGGCACCAATATAAAGCGCCGCGTAAGATGGTTGAAGAGCTGAG
CCGTCAGCTGAAACAAATCCACGACGTTGATTACACCCCGGCGGTGAAGAACGCGA
GCTTCCGTGACTGGGGCGAAGATCCGTTTGGCGGTGGCTGGAACAGCTGGAACAT
TGGTGTGAAGAGCTGGGAAGTTAAAGAGAAGATTGTGCACCCGATCGACAACTGCA
GCCTGTACATCTGCGGCGAAGCGTATAGCGATGGTCAAGGTTGGGTGGAGGGTGC
GCTGCAAACCGCGGACATTATGCTGAAGAAATTCATCGCGGTTGAGAGCAAAACCA
GCGTGAAGGAAGAGGCGATCCTGCTCGAG

Table S3. Summary for purification of HTAncLAAO2 and already reported LAAOs (HTAncLAAO and AncLAAOs) from 1L cultivation after HisTrap-HP purification.

Sample	Total protein (mg/L)	Specific activity (U/mg)	Total activity (U/L)
HTAncLAAO2	23.5	15.3	359
HTAncLAAO[a]	52.9	3.4	178
AncLAAON1[a]	50.7	12.8	651
AncLAAON4[a]	55.1	4.5	247
AncLAAON5[a]	78.4	2.1	166

[a]The parameters for HTAncLAAO, AncLAAON1, N4 and N5 were cited from the previous work [1, 2].

Table S4. Statistics of X-ray diffraction data collection of HTAncLAAO2 ligand free form.

	HTAncLAAO2
Space group	P4
Unit cell parameters	
a (Å)	180.9
b (Å)	180.9
c (Å)	81.4
α (degree)	90.0
β (degree)	90.0
γ (degree)	90.0
X-ray source	PF
	BL-5A
Wavelength (Å)	1.00
Resolution (Å)	48.5-2.20
	(2.32-2.20)
No. of reflections[a]	2741993
No. of unique reflections	133518
Completeness (%)	100.0 (99.9)
I/sig (I)	21.5 (5.3)
R_{merge}[b]	0.130 (0.651)
$CC_{1/2}$	0.999 (0.952)
B of Wilson plot (Å^2)	22.4
R[c]	0.169
R_{free}[d]	0.200
RMSD of geometry	
Bond length (Å)	0.009
Bond angles (deg)	1.52
Geometry	
Ramachandran outlier (%)	0.1
Ramachandran favored (%)	99.9
PDB entry	8JHE

a. Sigma cutoff was set to none (F > 0σF).

b. $R_{merge} = \Sigma_h \Sigma_i |I_i(h) - \langle I(h)\rangle| / \Sigma_h I(h)$, where $I_i(h)$ is the ith measurement of reflection *h*, and $\langle I(h)\rangle$ is the mean value of the symmetry-related reflection intensities. Values in brackets are for the shell of the highest resolution.

c. $R = \Sigma||F_o|-|F_c|| / \Sigma|F_o|$, where F_o and F_c are the observed and calculated structure factors used in the refinement, respectively.

d. R_{free} is the *R*-factor calculated using 5% of the reflections chosen at random and omitted from the refinement.

Table S5. Relative activity of HTAncLAAO2 and the variants toward 20 L-amino acids[a]

Substrate	Relative activity (%)			
	native	K304A	C505A	W220A
L-Phe	100 ± 0	100 ± 5.0	100 ± 1.3	100 ± 1.7
L-Leu	97.2 ± 0.8	98.2 ± 1.5	93.0 ± 1.4	138 ± 4.7
L-Met	85.3 ± 2.0	79.1 ± 1.8	71.1 ± 3.4	132 ± 3.2
L-Ile	91.5 ± 1.6	97.1 ± 1.4	86.7 ± 2.2	8.4 ± 1.6
L-Val	20.5 ± 1.6	29.0 ± 2.6	30.1 ± 1.4	N.D.
L-Tyr	15.3 ± 0.9	29.0 ± 1.6	31.4 ± 2.2	88.7 ± 1.0
L-Trp	3.2 ± 0.1	6.1 ± 3.1	6.8 ± 1.3	69.1 ± 2.8
LAla	N.D.	N.D.	N.D.	N.D.
L-His	N.D.	N.D.	N.D.	N.D.
L-Gln	N.D.	N.D.	N.D.	N.D.
L-Arg	N.D.	N.D.	N.D.	13.1 ± 4.2
L-Lys	N.D.	N.D.	N.D.	6.0 ± 1.4
L-Glu	N.D.	N.D.	N.D.	N.D.
L-Pro	N.D.	N.D.	N.D.	N.D.
L-Cys	N.D.	N.D.	N.D.	N.D.
L-Thr	N.D.	N.D.	N.D.	N.D.
L-Asn	N.D.	N.D.	N.D.	N.D.
L-Asp	N.D.	N.D.	N.D.	N.D.
L-Ser	N.D.	N.D.	N.D.	N.D.
Gly	N.D.	N.D.	N.D.	N.D.

[a]There is no activity toward 20 of D-amino acids.

Table S6. Enzyme kinetic parameters of HTAncLAAO2 variants toward five L-amino acids (L-Phe, L-Leu, L-Met, L-Ile and L-Trp)

Substrate		k_{cat} s^{-1}	K_m mM	k_{cat}/K_m s^{-1} mM^{-1}
L-Phe				
	native	14.5 ± 0.2	0.071 ± 0.004	204
	L320A	0.31 ± 0.0	7.5 ± 1.4	0.04
	Y383A	12.3 ± 0.6	26.5 ± 2.2	0.49
	W220A	14.6 ± 0.2	0.3 ± 0.01	45
L-Leu				
	native	13.2 ± 0.3	0.020 ± 0.002	661
	L320A	0.26 ± 0.0	0.6 ± 0.04	0.46
	Y383A	17.3 ± 0.2	1.6 ± 0.1	10.7
	W220A	21.8 ± 0.3	0.5 ± 0.03	41.1
L-Met				
	native	10.6 ± 0.1	0.031 ± 0.001	343
	L320A	0.20 ± 0.0	1.6 ± 0.2	0.13
	Y383A	10.6 ± 0.04	5.1 ± 0.1	2.1
	W220A	21.4 ± 0.2	0.1 ± 0.04	21.8
L-Ile				
	native	12.3 ± 0.0	0.081 ± 0.005	152
	L320A	0.09 ± 0.0	5.3 ± 0.1	0.02
	Y383A	10.3 ± 0.2	21.4 ± 1.1	0.48
	W220A	1.5 ± 0.0	1.0 ± 0.0	1.4
L-Trp				
	native	2.6 ± 0.1	15.4 ± 1.1	0.17
	L320A	0.004 ± 0.000	26.5 ± 1.8	$1.6E^{-4}$
	Y383A	1.2 ± 0.2	184 ± 38	$6.4E^{-3}$
	W220A	16.0 ± 0.2	7.5 ± 0.2	2.1

Table S7. Liquid chromatography (LC) condition and retention time for D,L-tryptophan derivatives (D,L-1a-c)[a]

Compound	Flow rate (mL/min)	Oven temperature (ºC)	Retention time (min)	
			D-enantiomer	L-enantiomer
D,L-1a	0.8	40	7.51	11.7
D,L-1b	0.8	40	6.67	11.6
D,L-1c	0.8	40	6.32	9.50

[a]The LC was performed by reverse phase HPLC on CROWNPAK-CR-I(+) column (150 mm × 3.0 mm × 5 μm, Daicel). Running buffer condition was 1.09% (w/v) $HClO_4$ and 5% (v/v) methanol for D,L-**1a** and **1c,** and 1.04% (w/v) $HClO_4$ and 10% (v/v) methanol for D,L-**1b**.

Table S8. A primer list to prepare HTAncLAAO2 variants.

	Sequences
W220A	5'- GGACGCGATTCCG**GCG**TACCTGAGCG -3'
L320A	5'- ACCCCGCGTCCG**GCG**TTCAAGGTGTTTACC -3'
Y383A	5'- ATGCTGCTGGCGAGC**GCG**GACGATGGTAGCAAC- 3'

Supplementary references

[1] Nakano, S., Kozuka, K., Minamino, Y., Karasuda, H., Hasebe, F., and Ito, S. (2020) Ancestral L-amino acid oxidases for deracemization and stereoinversion of amino acids, *Commun Chem 3*, 181.

[2] Ishida, C., Miyata, R., Hasebe, F., Miyata, A., Kumazawa, S., Ito, S., and Nakano, S. (2021) Reconstruction of Hyper-Thermostable Ancestral L-Amino Acid Oxidase to Perform Deracemization to D-Amino Acids, *ChemCatChem 13*, 5228-5235.
