# Supplementary material for: Structural and functional analysis of hyper-thermostable ancestral L-amino acid oxidase that can convert Trp derivatives to D-forms by chemoenzymatic reaction
Source: Commun Chem. 2023 Sep 22;6:200. doi: 10.1038/s42004-023-01005-1 (PMC10517122; doi:10.1038/s42004-023-01005-1)
Supplement: Supplementary file 3 — Description of Additional Supplementary Files [file 42004_2023_1005_MOESM3_ESM.pdf]

Description of Additional Supplementary Files

File name: Supplementary Data 1

Description: Validation report of PDB ID: 8JHE
