# Supplementary material for: Structural and functional analysis of hyper-thermostable ancestral L-amino acid oxidase that can convert Trp derivatives to D-forms by chemoenzymatic reaction
Source: Commun Chem. 2023 Sep 22;6:200. doi: 10.1038/s42004-023-01005-1 (PMC10517122; doi:10.1038/s42004-023-01005-1)
Supplement: Supplementary file 4 — Supplementary Data 1 [file 42004_2023_1005_MOESM4_ESM.pdf]

Full wwPDB X-ray Structure Validation Report (i)

Jun 3, 2023 – 05:01 PM JST

PDB ID	:	8JHE
Title	:	Hyper-thermostable ancestral L-amino acid oxidase 2 (HTAncLAAO2)
Deposited on	:	2023-05-23
Resolution	:	2.20 Å(reported)

This wwPDB validation report is for manuscript review

This is a Full wwPDB X-ray Structure Validation Report.

This report is produced by the wwPDB biocuration pipeline after annotation of the structure.

We welcome your comments at *validation@mail.wwpdb.org*
A user guide is available at
https://www.wwpdb.org/validation/2017/XrayValidationReportHelp
with specific help available everywhere you see the (i) symbol.

The types of validation reports are described at
http://www.wwpdb.org/validation/2017/FAQs#types.

The following versions of software and data (see references (i)) were used in the production of this report:

MolProbity	:	4.02b-467
Mogul	:	1.8.5 (274361), CSD as541be (2020)
Xtriage (Phenix)	:	1.13
EDS	:	2.33
buster-report	:	1.1.7 (2018)
Percentile statistics	:	20191225.v01 (using entries in the PDB archive December 25th 2019)
Refmac	:	5.8.0158
CCP4	:	7.0.044 (Gargrove)
Ideal geometry (proteins)	:	Engh & Huber (2001)
Ideal geometry (DNA, RNA)	:	Parkinson et al. (1996)

For Manuscript Review

1 Overall quality at a glance (i)

The following experimental techniques were used to determine the structure:
X-RAY DIFFRACTION

The reported resolution of this entry is 2.20 Å.

Percentile scores (ranging between 0-100) for global validation metrics of the entry are shown in the following graphic. The table shows the number of entries on which the scores are based.

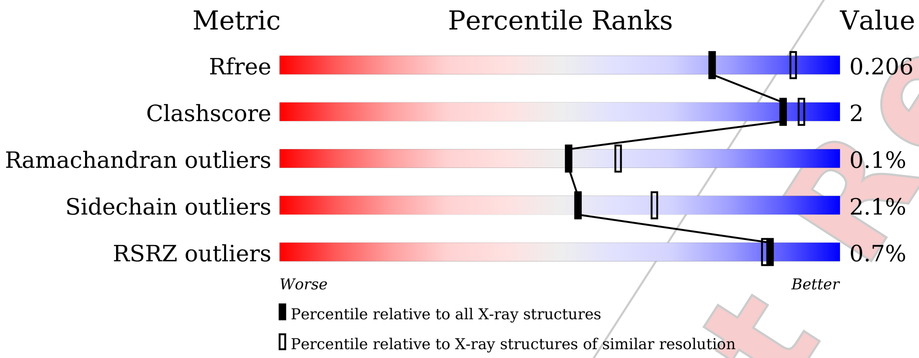

Metric	Whole archive (#Entries)	Similar resolution (#Entries, resolution range(Å))
R_{free}	130704	4898 (2.20-2.20)
Clashscore	141614	5594 (2.20-2.20)
Ramachandran outliers	138981	5503 (2.20-2.20)
Sidechain outliers	138945	5504 (2.20-2.20)
RSRZ outliers	127900	4800 (2.20-2.20)

The table below summarises the geometric issues observed across the polymeric chains and their fit to the electron density. The red, orange, yellow and green segments of the lower bar indicate the fraction of residues that contain outliers for >=3, 2, 1 and 0 types of geometric quality criteria respectively. A grey segment represents the fraction of residues that are not modelled. The numeric value for each fraction is indicated below the corresponding segment, with a dot representing fractions <=5% The upper red bar (where present) indicates the fraction of residues that have poor fit to the electron density. The numeric value is given above the bar.

Mol	Chain	Length	Quality of chain
1	A	555	% / 85% 6% 8%
1	B	555	87% • 9%
1	C	555	86% 5% 9%
1	D	555	% / 85% 6% 9%

For Manuscript Review

2 Entry composition (i)

There are 3 unique types of molecules in this entry. The entry contains 17963 atoms, of which 0 are hydrogens and 0 are deuteriums.

In the tables below, the ZeroOcc column contains the number of atoms modelled with zero occupancy, the AltConf column contains the number of residues with at least one atom in alternate conformation and the Trace column contains the number of residues modelled with at most 2 atoms.

- Molecule 1 is a protein called Hyper thermostable ancestral L-amino acid oxidase.

Mol	Chain	Residues	Atoms					ZeroOcc	AltConf	Trace
1	A	508	Total 4038	C 2593	N 666	O 769	S 10	0	0	0
1	B	504	Total 4010	C 2577	N 661	O 762	S 10	0	0	0
1	C	504	Total 4010	C 2577	N 661	O 762	S 10	0	0	0
1	D	504	Total 4010	C 2577	N 661	O 762	S 10	0	0	0

- Molecule 2 is FLAVIN-ADENINE DINUCLEOTIDE (three-letter code: FAD) (formula: $C_{27}H_{33}N_{9}O_{15}P_{2}$) (labeled as "Ligand of Interest" by depositor).

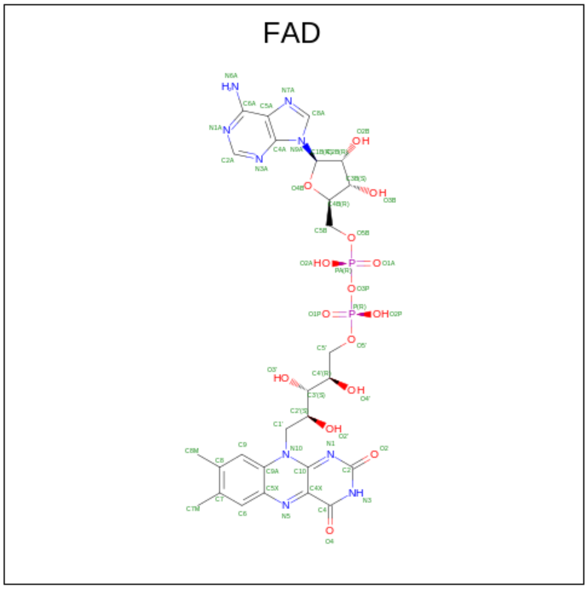

Mol	Chain	Residues	Atoms					ZeroOcc	AltConf
2	A	1	Total 53	C 27	N 9	O 15	P 2	0	0
2	B	1	Total 53	C 27	N 9	O 15	P 2	0	0

Continued on next page...

For Manuscript Review

Continued from previous page...

Mol	Chain	Residues	Atoms					ZeroOcc	AltConf
			Total	C	N	O	P		
2	C	1	53	27	9	15	2	0	0
2	D	1	53	27	9	15	2	0	0

- Molecule 3 is water.

Mol	Chain	Residues	Atoms		ZeroOcc	AltConf
			Total	O		
3	A	415	415	415	0	0
3	B	427	427	427	0	0
3	C	407	407	407	0	0
3	D	434	434	434	0	0

For Manuscript Review

3 Residue-property plots ⓘ

These plots are drawn for all protein, RNA, DNA and oligosaccharide chains in the entry. The first graphic for a chain summarises the proportions of the various outlier classes displayed in the second graphic. The second graphic shows the sequence view annotated by issues in geometry and electron density. Residues are color-coded according to the number of geometric quality criteria for which they contain at least one outlier: green = 0, yellow = 1, orange = 2 and red = 3 or more. A red dot above a residue indicates a poor fit to the electron density (RSRZ > 2). Stretches of 2 or more consecutive residues without any outlier are shown as a green connector. Residues present in the sample, but not in the model, are shown in grey.

• Molecule 1: Hyper thermostable ancestral L-amino acid oxidase

Chain A:

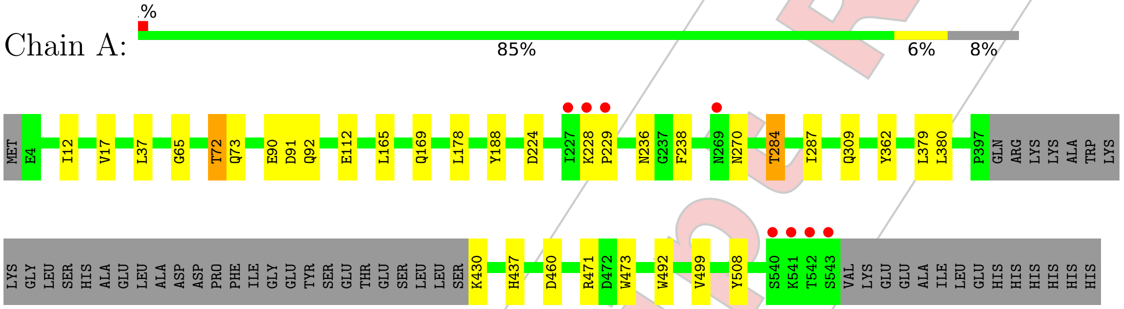

• Molecule 1: Hyper thermostable ancestral L-amino acid oxidase

Chain B:

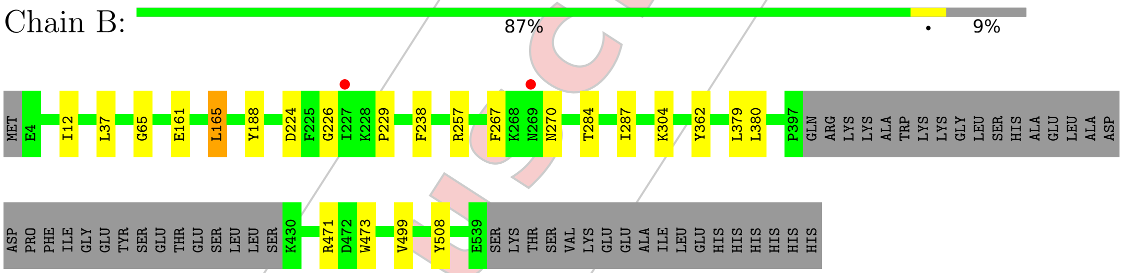

• Molecule 1: Hyper thermostable ancestral L-amino acid oxidase

Chain C:

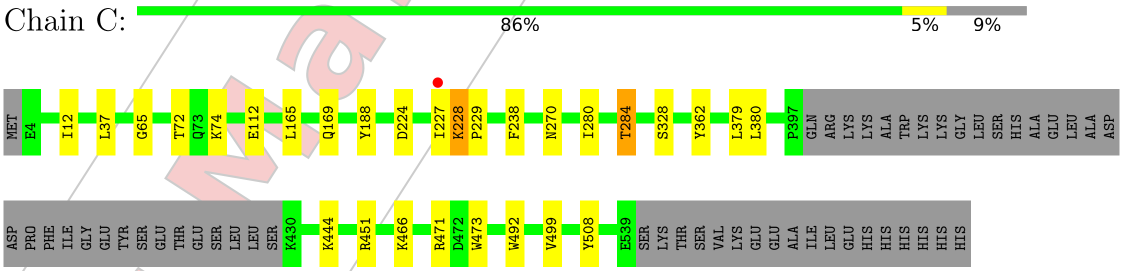

• Molecule 1: Hyper thermostable ancestral L-amino acid oxidase

Chain D:

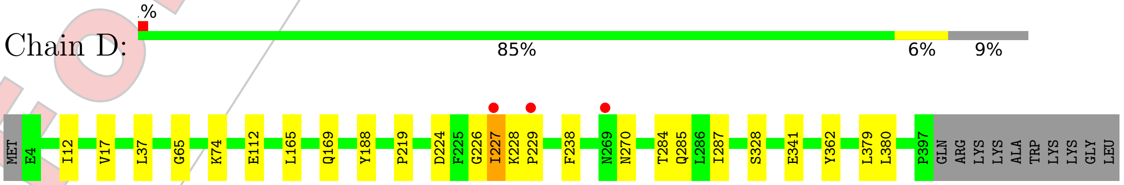

For Manuscript Review

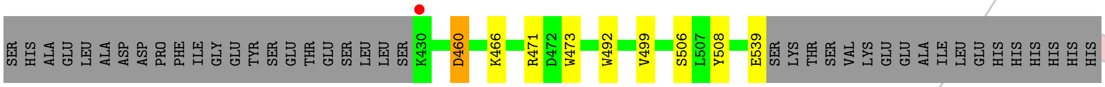

For Manuscript Review

4 Data and refinement statistics (i)

Property	Value	Source
Space group	P 4	Depositor
Cell constants<br>a, b, c, α, β, γ	180.90Å 180.90Å 81.41Å<br>90.00° 90.00° 90.00°	Depositor
Resolution (Å)	48.50 – 2.20<br>48.45 – 2.20	Depositor<br>EDS
% Data completeness<br>(in resolution range)	100.0 (48.50-2.20)<br>100.0 (48.45-2.20)	Depositor<br>EDS
R_{merge}	(Not available)	Depositor
R_{sym}	(Not available)	Depositor
$< I/\sigma(I) >$ [1]	4.23 (at 2.20Å)	Xtriage
Refinement program	REFMAC 5.8.0267	Depositor
R, R_{free}	0.167 , 0.200<br>0.174 , 0.206	Depositor<br>DCC
R_{free} test set	6626 reflections (4.96%)	wwPDB-VP
Wilson B-factor (Å^2)	25.7	Xtriage
Anisotropy	0.022	Xtriage
Bulk solvent k_{sol}(e/Å^3), B_{sol}(Å^2)	0.34 , 50.0	EDS
L-test for twinning[2]	$< \|L\| > = 0.52$, $< L^2 > = 0.35$	Xtriage
Estimated twinning fraction	0.017 for h,-k,-l	Xtriage
F_o,F_c correlation	0.96	EDS
Total number of atoms	17963	wwPDB-VP
Average B, all atoms (Å^2)	29.0	wwPDB-VP

Xtriage's analysis on translational NCS is as follows: *The analyses of the Patterson function reveals a significant off-origin peak that is 38.25 % of the origin peak, indicating pseudo-translational symmetry. The chance of finding a peak of this or larger height randomly in a structure without pseudo-translational symmetry is equal to 3.7912e-04. The detected translational NCS is most likely also responsible for the elevated intensity ratio.*

[1] Intensities estimated from amplitudes.
[2] Theoretical values of $< |L| >$, $< L^2 >$ for acentric reflections are 0.5, 0.333 respectively for untwinned datasets, and 0.375, 0.2 for perfectly twinned datasets.

For Manuscript Review

5 Model quality (i)

5.1 Standard geometry (i)

Bond lengths and bond angles in the following residue types are not validated in this section: FAD

The Z score for a bond length (or angle) is the number of standard deviations the observed value is removed from the expected value. A bond length (or angle) with $|Z| > 5$ is considered an outlier worth inspection. RMSZ is the root-mean-square of all Z scores of the bond lengths (or angles).

Mol	Chain	Bond lengths		Bond angles	
		RMSZ	#$|Z|$ >5	RMSZ	#$|Z|$ >5
1	A	0.69	0/4139	0.80	0/5619
1	B	0.70	0/4111	0.80	0/5582
1	C	0.70	0/4111	0.81	2/5582 (0.0%)
1	D	0.71	0/4111	0.80	0/5582
All	All	0.70	0/16472	0.80	2/22365 (0.0%)

Chiral center outliers are detected by calculating the chiral volume of a chiral center and verifying if the center is modelled as a planar moiety or with the opposite hand.A planarity outlier is detected by checking planarity of atoms in a peptide group, atoms in a mainchain group or atoms of a sidechain that are expected to be planar.

Mol	Chain	#Chirality outliers	#Planarity outliers
1	A	0	1
1	B	0	1
1	C	0	1
1	D	0	1
All	All	0	4

There are no bond length outliers.

All (2) bond angle outliers are listed below:

Mol	Chain	Res	Type	Atoms	Z	Observed(°)	Ideal(°)
1	C	451	ARG	NE-CZ-NH2	-7.50	116.55	120.30
1	C	451	ARG	NE-CZ-NH1	6.30	123.45	120.30

There are no chirality outliers.

All (4) planarity outliers are listed below:

For Manuscript Review

Mol	Chain	Res	Type	Group
1	A	379	LEU	Peptide
1	B	379	LEU	Peptide
1	C	379	LEU	Peptide
1	D	379	LEU	Peptide

5.2 Too-close contacts (i)

In the following table, the Non-H and H(model) columns list the number of non-hydrogen atoms and hydrogen atoms in the chain respectively. The H(added) column lists the number of hydrogen atoms added and optimized by MolProbity. The Clashes column lists the number of clashes within the asymmetric unit, whereas Symm-Clashes lists symmetry-related clashes.

Mol	Chain	Non-H	H(model)	H(added)	Clashes	Symm-Clashes
1	A	4038	0	3953	18	0
1	B	4010	0	3923	11	0
1	C	4010	0	3924	14	0
1	D	4010	0	3924	17	0
2	A	53	0	31	2	0
2	B	53	0	31	2	0
2	C	53	0	31	3	0
2	D	53	0	31	3	0
3	A	415	0	0	3	0
3	B	427	0	0	0	1
3	C	407	0	0	2	0
3	D	434	0	0	3	1
All	All	17963	0	15848	59	1

The all-atom clashscore is defined as the number of clashes found per 1000 atoms (including hydrogen atoms). The all-atom clashscore for this structure is 2.

All (59) close contacts within the same asymmetric unit are listed below, sorted by their clash magnitude.

Atom-1	Atom-2	Interatomic distance (Å)	Clash overlap (Å)
1:D:226:GLY:O	1:D:227:ILE:O	1.98	0.82
1:D:112:GLU:OE1	3:D:701:HOH:O	2.00	0.79
1:C:112:GLU:OE2	3:C:701:HOH:O	2.01	0.76
1:D:460:ASP:OD2	3:D:702:HOH:O	2.05	0.74
1:A:112:GLU:OE2	3:A:701:HOH:O	2.05	0.73
1:A:72:THR:HG22	1:A:73:GLN:HG3	1.74	0.70
1:D:539:GLU:C	3:D:1007:HOH:O	2.33	0.67
1:A:284:THR:HG21	3:A:708:HOH:O	2.01	0.60

Continued on next page...

For Manuscript Review

Continued from previous page...

Atom-1	Atom-2	Interatomic distance (Å)	Clash overlap (Å)
1:B:65:GLY:HA2	2:B:601:FAD:C4X	2.32	0.58
1:A:72:THR:CG2	1:A:73:GLN:HG3	2.35	0.56
1:D:65:GLY:HA2	2:D:601:FAD:C4X	2.35	0.56
1:C:65:GLY:HA2	2:C:601:FAD:C4X	2.35	0.55
1:D:65:GLY:HA2	2:D:601:FAD:N5	2.22	0.54
1:A:65:GLY:HA2	2:A:601:FAD:C4X	2.38	0.53
1:C:169:GLN:HG2	1:C:492:TRP:CE3	2.44	0.52
1:D:169:GLN:HG2	1:D:492:TRP:CE3	2.46	0.51
1:D:362:TYR:HB2	1:D:380:LEU:HB3	1.93	0.51
1:C:280:ILE:HD12	1:C:280:ILE:N	2.26	0.51
1:C:65:GLY:HA2	2:C:601:FAD:N5	2.26	0.50
1:A:169:GLN:HG2	1:A:492:TRP:CE3	2.46	0.50
1:A:362:TYR:HB2	1:A:380:LEU:HB3	1.93	0.50
1:B:362:TYR:HB2	1:B:380:LEU:HB3	1.93	0.49
1:A:284:THR:CG2	3:A:708:HOH:O	2.61	0.49
1:C:362:TYR:HB2	1:C:380:LEU:HB3	1.95	0.48
1:C:328:SER:HB3	1:C:466:LYS:HE3	1.96	0.48
1:B:65:GLY:HA2	2:B:601:FAD:N5	2.29	0.48
1:B:471:ARG:HG2	1:B:473:TRP:CZ2	2.50	0.46
1:D:328:SER:HB3	1:D:466:LYS:HE3	1.97	0.46
1:A:65:GLY:HA2	2:A:601:FAD:N5	2.32	0.45
1:C:471:ARG:HG2	1:C:473:TRP:CZ2	2.52	0.45
1:D:471:ARG:HG2	1:D:473:TRP:CZ2	2.53	0.43
1:B:267:PHE:CG	1:B:304:LYS:HE3	2.53	0.43
1:A:178:LEU:HD23	1:A:178:LEU:HA	1.94	0.43
1:D:12:ILE:O	1:D:37:LEU:HA	2.19	0.43
1:D:65:GLY:HA2	2:D:601:FAD:C5X	2.49	0.43
1:A:12:ILE:O	1:A:37:LEU:HA	2.19	0.42
1:B:499:VAL:O	1:B:508:TYR:HA	2.19	0.42
1:A:471:ARG:HG2	1:A:473:TRP:CZ2	2.55	0.42
1:C:12:ILE:O	1:C:37:LEU:HA	2.20	0.42
1:D:285:GLN:HG3	1:D:506:SER:HB3	2.02	0.42
1:C:284:THR:HG22	3:C:927:HOH:O	2.19	0.42
1:A:236:ASN:OD1	1:A:430:LYS:HE2	2.20	0.42
1:C:224:ASP:O	1:C:229:PRO:HB3	2.20	0.42
1:D:224:ASP:O	1:D:229:PRO:HB3	2.20	0.42
1:B:165:LEU:HD23	1:B:165:LEU:HA	1.93	0.41
1:D:165:LEU:HD12	1:D:219:PRO:HG2	2.02	0.41
1:A:499:VAL:O	1:A:508:TYR:HA	2.20	0.41
1:D:499:VAL:O	1:D:508:TYR:HA	2.20	0.41
1:D:287:ILE:HG12	1:D:508:TYR:HB2	2.03	0.41

Continued on next page...

For Manuscript Review

Continued from previous page...

Atom-1	Atom-2	Interatomic distance (Å)	Clash overlap (Å)
1:A:91:ASP:O	1:A:92:GLN:C	2.59	0.41
1:B:287:ILE:HG12	1:B:508:TYR:HB2	2.02	0.41
1:C:499:VAL:O	1:C:508:TYR:HA	2.21	0.41
1:A:437:HIS:CD2	1:B:257:ARG:HD3	2.55	0.41
1:A:287:ILE:HG12	1:A:508:TYR:HB2	2.03	0.40
1:B:224:ASP:O	1:B:229:PRO:HB3	2.22	0.40
1:C:65:GLY:HA2	2:C:601:FAD:C5X	2.51	0.40
1:B:12:ILE:O	1:B:37:LEU:HA	2.21	0.40
1:C:228:LYS:HA	1:C:228:LYS:HD3	1.78	0.40
1:A:224:ASP:O	1:A:229:PRO:HB3	2.21	0.40

All (1) symmetry-related close contacts are listed below. The label for Atom-2 includes the symmetry operator and encoded unit-cell translations to be applied.

Atom-1	Atom-2	Interatomic distance (Å)	Clash overlap (Å)
3:B:1059:HOH:O	3:D:1132:HOH:O[1_554]	2.13	0.07

5.3 Torsion angles (i)

5.3.1 Protein backbone (i)

In the following table, the Percentiles column shows the percent Ramachandran outliers of the chain as a percentile score with respect to all X-ray entries followed by that with respect to entries of similar resolution.

The Analysed column shows the number of residues for which the backbone conformation was analysed, and the total number of residues.

Mol	Chain	Analysed	Favoured	Allowed	Outliers	Percentiles	
1	A	504/555 (91%)	488 (97%)	16 (3%)	0	100	100
1	B	500/555 (90%)	484 (97%)	15 (3%)	1 (0%)	47	55
1	C	500/555 (90%)	485 (97%)	15 (3%)	0	100	100
1	D	500/555 (90%)	482 (96%)	17 (3%)	1 (0%)	47	55
All	All	2004/2220 (90%)	1939 (97%)	63 (3%)	2 (0%)	51	60

All (2) Ramachandran outliers are listed below:

For Manuscript Review

Mol	Chain	Res	Type
1	D	227	ILE
1	B	226	GLY

5.3.2 Protein sidechains (i)

In the following table, the Percentiles column shows the percent sidechain outliers of the chain as a percentile score with respect to all X-ray entries followed by that with respect to entries of similar resolution.

The Analysed column shows the number of residues for which the sidechain conformation was analysed, and the total number of residues.

Mol	Chain	Analysed	Rotameric	Outliers	Percentiles	
1	A	437/478 (91%)	426 (98%)	11 (2%)	47	60
1	B	433/478 (91%)	427 (99%)	6 (1%)	67	80
1	C	433/478 (91%)	423 (98%)	10 (2%)	50	63
1	D	433/478 (91%)	424 (98%)	9 (2%)	53	67
All	All	1736/1912 (91%)	1700 (98%)	36 (2%)	53	67

All (36) residues with a non-rotameric sidechain are listed below:

Mol	Chain	Res	Type
1	A	17	VAL
1	A	72	THR
1	A	90	GLU
1	A	165	LEU
1	A	188	TYR
1	A	228	LYS
1	A	238	PHE
1	A	270	ASN
1	A	284	THR
1	A	309	GLN
1	A	460	ASP
1	B	161	GLU
1	B	165	LEU
1	B	188	TYR
1	B	238	PHE
1	B	270	ASN
1	B	284	THR
1	C	72	THR
1	C	74	LYS
1	C	165	LEU

Continued on next page...

For Manuscript Review

Continued from previous page...

Mol	Chain	Res	Type
1	C	188	TYR
1	C	227	ILE
1	C	228	LYS
1	C	238	PHE
1	C	270	ASN
1	C	284	THR
1	C	444	LYS
1	D	17	VAL
1	D	74	LYS
1	D	188	TYR
1	D	228	LYS
1	D	238	PHE
1	D	270	ASN
1	D	284	THR
1	D	341	GLU
1	D	460	ASP

Sometimes sidechains can be flipped to improve hydrogen bonding and reduce clashes. All (3) such sidechains are listed below:

Mol	Chain	Res	Type
1	A	121	ASN
1	C	300	ASN
1	D	300	ASN

5.3.3 RNA (i)

There are no RNA molecules in this entry.

5.4 Non-standard residues in protein, DNA, RNA chains (i)

There are no non-standard protein/DNA/RNA residues in this entry.

5.5 Carbohydrates (i)

There are no monosaccharides in this entry.

5.6 Ligand geometry (i)

4 ligands are modelled in this entry.

For Manuscript Review

In the following table, the Counts columns list the number of bonds (or angles) for which Mogul statistics could be retrieved, the number of bonds (or angles) that are observed in the model and the number of bonds (or angles) that are defined in the Chemical Component Dictionary. The Link column lists molecule types, if any, to which the group is linked. The Z score for a bond length (or angle) is the number of standard deviations the observed value is removed from the expected value. A bond length (or angle) with $|Z| > 2$ is considered an outlier worth inspection. RMSZ is the root-mean-square of all Z scores of the bond lengths (or angles).

Mol	Type	Chain	Res	Link	Bond lengths			Bond angles		
					Counts	RMSZ	$\#\|Z\|>2$	Counts	RMSZ	$\#\|Z\|>2$
2	FAD	A	601	-	53,58,58	0.73	2 (3%)	68,89,89	0.93	2 (2%)
2	FAD	D	601	-	53,58,58	0.72	1 (1%)	68,89,89	0.91	2 (2%)
2	FAD	C	601	-	53,58,58	0.69	0	68,89,89	0.81	2 (2%)
2	FAD	B	601	-	53,58,58	0.70	2 (3%)	68,89,89	0.82	2 (2%)

In the following table, the Chirals column lists the number of chiral outliers, the number of chiral centers analysed, the number of these observed in the model and the number defined in the Chemical Component Dictionary. Similar counts are reported in the Torsion and Rings columns. '-' means no outliers of that kind were identified.

Mol	Type	Chain	Res	Link	Chirals	Torsions	Rings
2	FAD	A	601	-	-	3/30/50/50	0/6/6/6
2	FAD	D	601	-	-	3/30/50/50	0/6/6/6
2	FAD	C	601	-	-	5/30/50/50	0/6/6/6
2	FAD	B	601	-	-	2/30/50/50	0/6/6/6

All (5) bond length outliers are listed below:

Mol	Chain	Res	Type	Atoms	Z	Observed(Å)	Ideal(Å)
2	A	601	FAD	C1'-C2'	-2.70	1.48	1.52
2	D	601	FAD	C1'-C2'	-2.35	1.49	1.52
2	B	601	FAD	C8A-N7A	-2.11	1.30	1.34
2	B	601	FAD	C1'-C2'	-2.10	1.49	1.52
2	A	601	FAD	C8A-N7A	-2.01	1.31	1.34

All (8) bond angle outliers are listed below:

Mol	Chain	Res	Type	Atoms	Z	Observed(^{o})	Ideal(^{o})
2	A	601	FAD	P-O3P-PA	2.39	141.02	132.83
2	C	601	FAD	C5A-C6A-N6A	2.27	123.80	120.35
2	C	601	FAD	C1'-C2'-C3'	-2.20	103.62	109.79
2	D	601	FAD	P-O3P-PA	2.19	140.33	132.83

Continued on next page...

For Manuscript Review

Continued from previous page...

Mol	Chain	Res	Type	Atoms	Z	Observed(°)	Ideal(°)
2	D	601	FAD	O3'-C3'-C2'	2.14	113.99	108.81
2	A	601	FAD	C1'-C2'-C3'	-2.09	103.94	109.79
2	B	601	FAD	C1'-C2'-C3'	-2.06	104.03	109.79
2	B	601	FAD	C5A-C6A-N6A	2.02	123.42	120.35

There are no chirality outliers.

All (13) torsion outliers are listed below:

Mol	Chain	Res	Type	Atoms
2	D	601	FAD	PA-O3P-P-O1P
2	A	601	FAD	PA-O3P-P-O5'
2	B	601	FAD	PA-O3P-P-O5'
2	C	601	FAD	PA-O3P-P-O5'
2	D	601	FAD	PA-O3P-P-O5'
2	A	601	FAD	PA-O3P-P-O1P
2	C	601	FAD	PA-O3P-P-O1P
2	C	601	FAD	C2'-C3'-C4'-O4'
2	A	601	FAD	O4B-C4B-C5B-O5B
2	C	601	FAD	O4B-C4B-C5B-O5B
2	C	601	FAD	C2'-C3'-C4'-C5'
2	B	601	FAD	O4B-C4B-C5B-O5B
2	D	601	FAD	O4B-C4B-C5B-O5B

There are no ring outliers.

4 monomers are involved in 10 short contacts:

Mol	Chain	Res	Type	Clashes	Symm-Clashes
2	A	601	FAD	2	0
2	D	601	FAD	3	0
2	C	601	FAD	3	0
2	B	601	FAD	2	0

The following is a two-dimensional graphical depiction of Mogul quality analysis of bond lengths, bond angles, torsion angles, and ring geometry for all instances of the Ligand of Interest. In addition, ligands with molecular weight > 250 and outliers as shown on the validation Tables will also be included. For torsion angles, if less then 5% of the Mogul distribution of torsion angles is within 10 degrees of the torsion angle in question, then that torsion angle is considered an outlier. Any bond that is central to one or more torsion angles identified as an outlier by Mogul will be highlighted in the graph. For rings, the root-mean-square deviation (RMSD) between the ring in question and similar rings identified by Mogul is calculated over all ring torsion angles. If the average RMSD is greater than 60 degrees and the minimal RMSD between the ring in question and any Mogul-identified rings is also greater than 60 degrees, then that ring is considered an outlier.

For Manuscript Review

The outliers are highlighted in purple. The color gray indicates Mogul did not find sufficient equivalents in the CSD to analyse the geometry.

Ligand FAD A 601

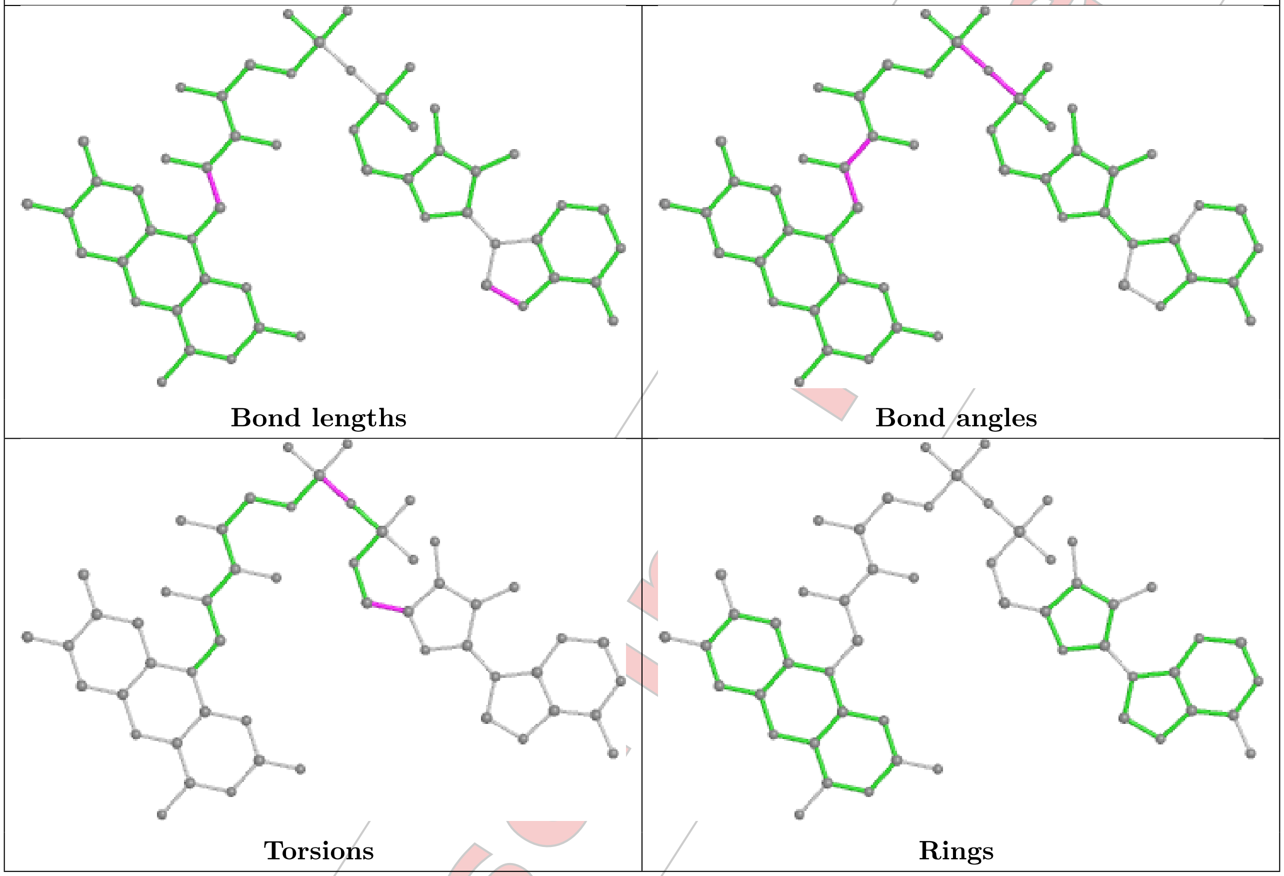

For Manuscript Review

Ligand FAD D 601

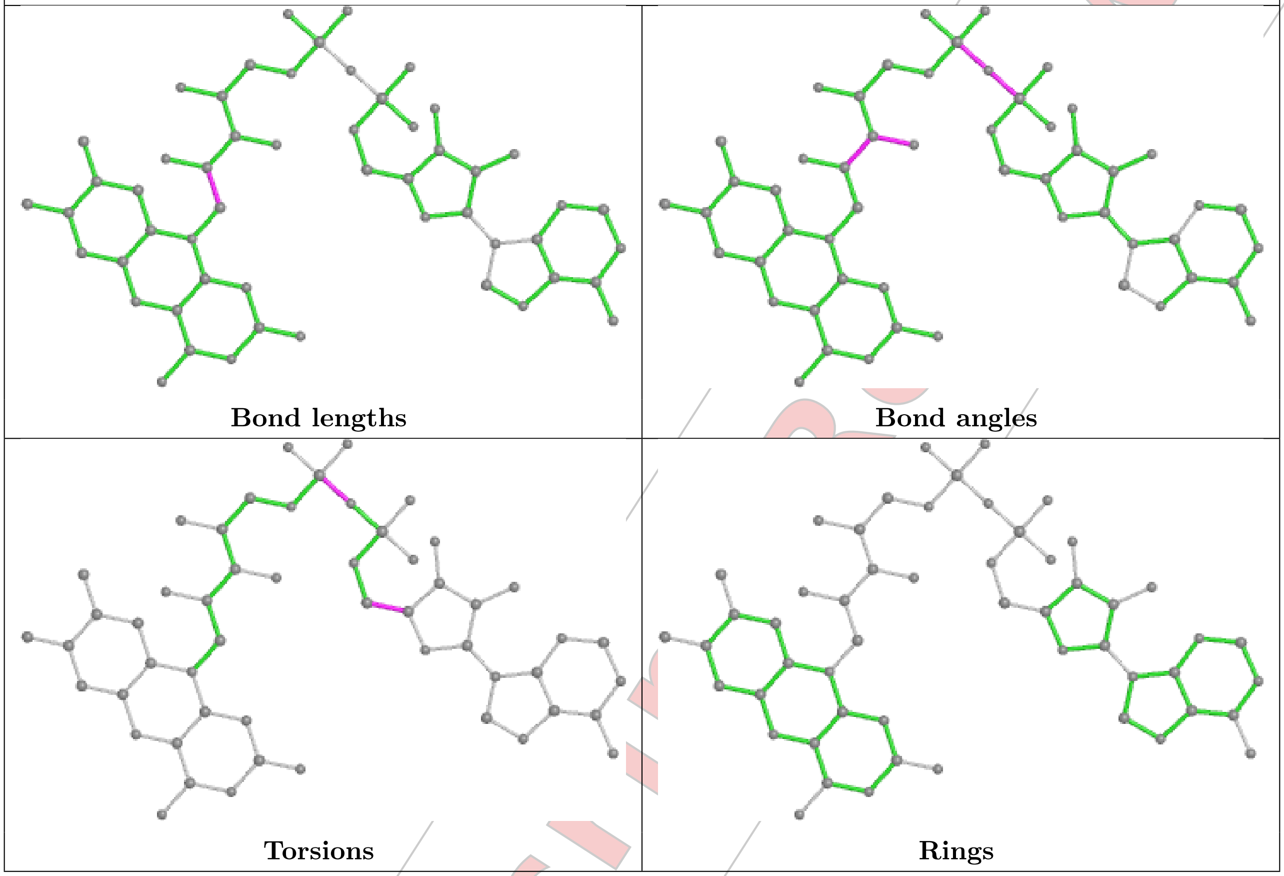

For Manuscript Review

Ligand FAD C 601

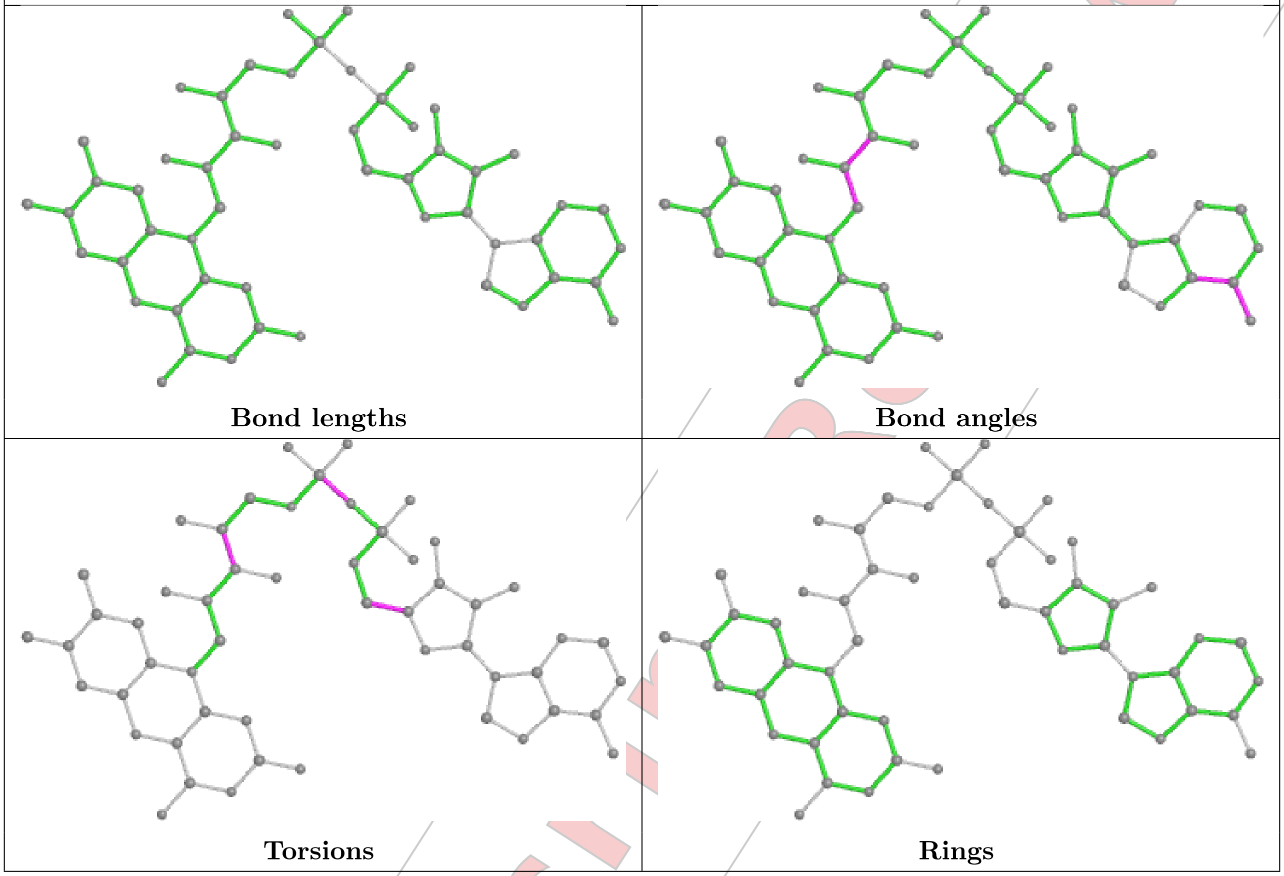

For Manuscript Review

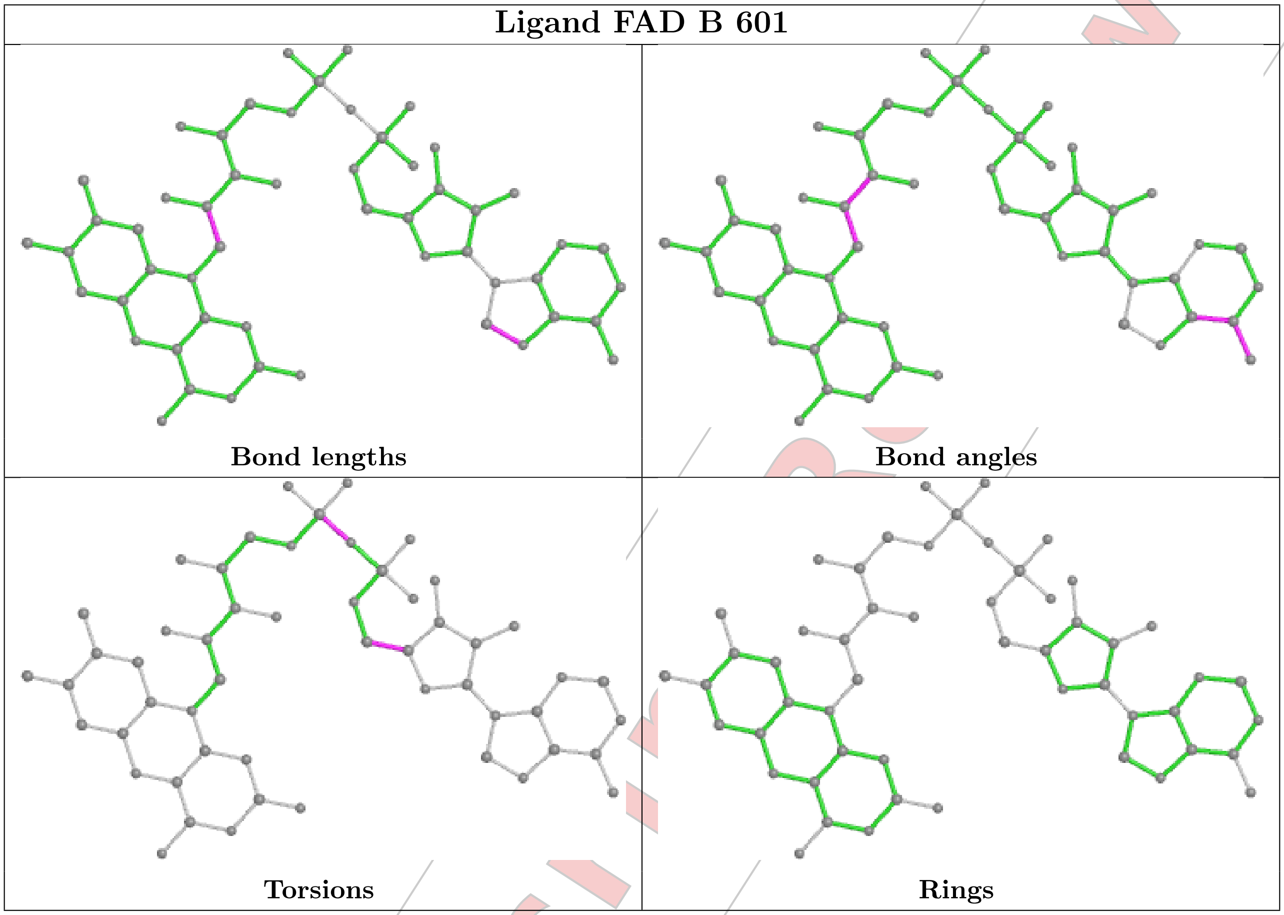

5.7 Other polymers (i)

There are no such residues in this entry.

5.8 Polymer linkage issues (i)

There are no chain breaks in this entry.

For Manuscript Review

6 Fit of model and data (i)

6.1 Protein, DNA and RNA chains (i)

In the following table, the column labelled '#RSRZ> 2' contains the number (and percentage) of RSRZ outliers, followed by percent RSRZ outliers for the chain as percentile scores relative to all X-ray entries and entries of similar resolution. The OWAB column contains the minimum, median, 95th percentile and maximum values of the occupancy-weighted average B-factor per residue. The column labelled 'Q< 0.9' lists the number of (and percentage) of residues with an average occupancy less than 0.9.

Mol	Chain	Analysed	<RSRZ>	#RSRZ>2			OWAB(Å^2)	Q<0.9
1	A	508/555 (91%)	-0.46	8 (1%)	72	70	18, 26, 51, 84	0
1	B	504/555 (90%)	-0.47	2 (0%)	92	91	18, 25, 49, 73	0
1	C	504/555 (90%)	-0.43	1 (0%)	95	94	18, 26, 48, 83	0
1	D	504/555 (90%)	-0.36	4 (0%)	86	85	18, 27, 50, 78	0
All	All	2020/2220 (90%)	-0.43	15 (0%)	87	86	18, 26, 50, 84	0

All (15) RSRZ outliers are listed below:

Mol	Chain	Res	Type	RSRZ
1	C	227	ILE	4.8
1	B	227	ILE	4.0
1	A	227	ILE	3.7
1	A	542	THR	3.4
1	D	430	LYS	3.1
1	D	269	ASN	2.9
1	A	269	ASN	2.8
1	A	229	PRO	2.8
1	A	541	LYS	2.8
1	A	543	SER	2.8
1	D	227	ILE	2.6
1	A	540	SER	2.6
1	B	269	ASN	2.6
1	D	229	PRO	2.1
1	A	228	LYS	2.1

6.2 Non-standard residues in protein, DNA, RNA chains (i)

There are no non-standard protein/DNA/RNA residues in this entry.

For Manuscript Review

6.3 Carbohydrates (i)

There are no monosaccharides in this entry.

6.4 Ligands (i)

In the following table, the Atoms column lists the number of modelled atoms in the group and the number defined in the chemical component dictionary. The B-factors column lists the minimum, median, 95th percentile and maximum values of B factors of atoms in the group. The column labelled 'Q< 0.9' lists the number of atoms with occupancy less than 0.9.

Mol	Type	Chain	Res	Atoms	RSCC	RSR	B-factors(Å^2)	Q<0.9
2	FAD	D	601	53/53	0.96	0.10	18,23,30,35	0
2	FAD	B	601	53/53	0.97	0.09	16,22,28,29	0
2	FAD	C	601	53/53	0.97	0.09	18,23,30,32	0
2	FAD	A	601	53/53	0.97	0.09	17,22,28,30	0

The following is a graphical depiction of the model fit to experimental electron density of all instances of the Ligand of Interest. In addition, ligands with molecular weight > 250 and outliers as shown on the geometry validation Tables will also be included. Each fit is shown from different orientation to approximate a three-dimensional view.

Electron density around FAD D 601:

$2mF_o$-DF_c (at 0.7 rmsd) in gray
mF_o-DF_c (at 3 rmsd) in purple (negative) and green (positive)

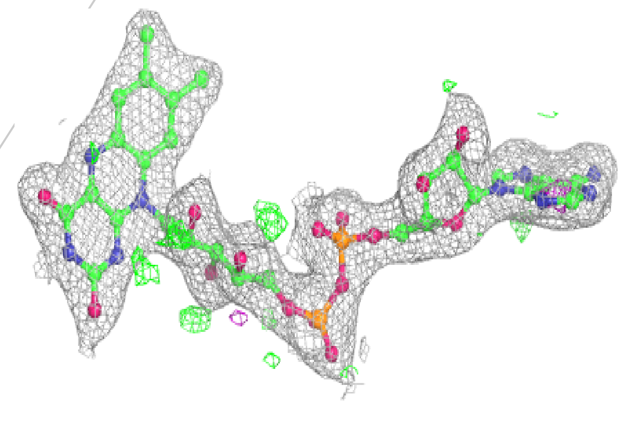

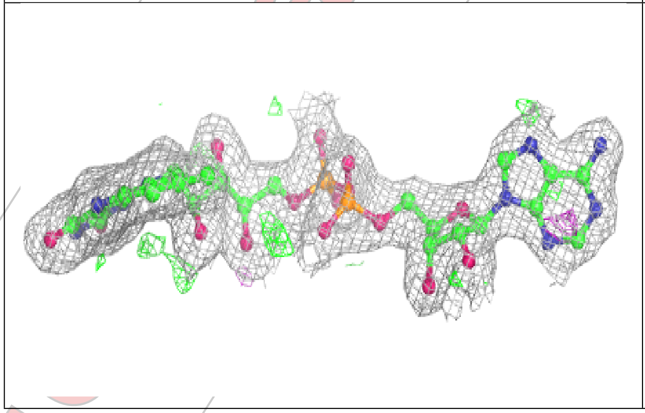

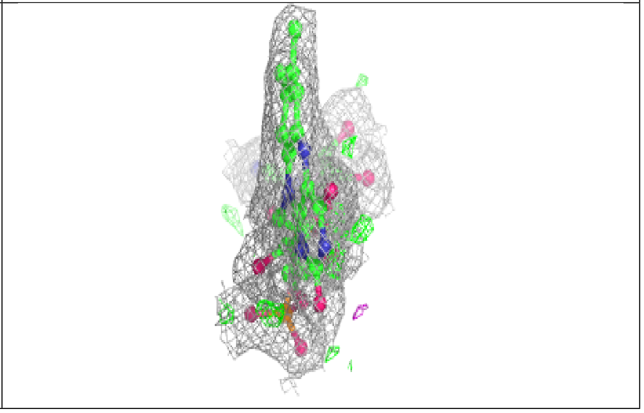

For Manuscript Review

Electron density around FAD B 601:

$2mF_o\text{-}DF_c$ (at 0.7 rmsd) in gray
$mF_o\text{-}DF_c$ (at 3 rmsd) in purple (negative)
and green (positive)

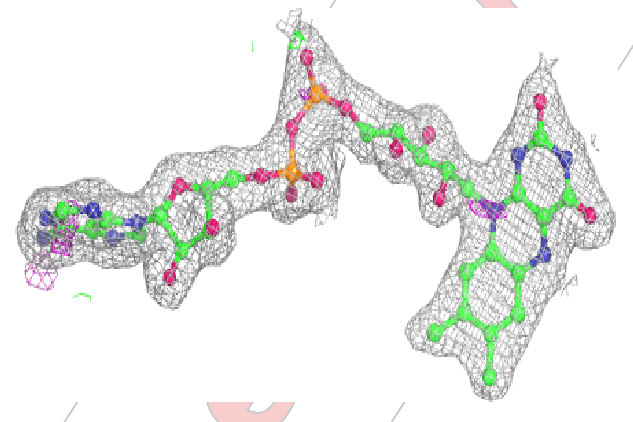

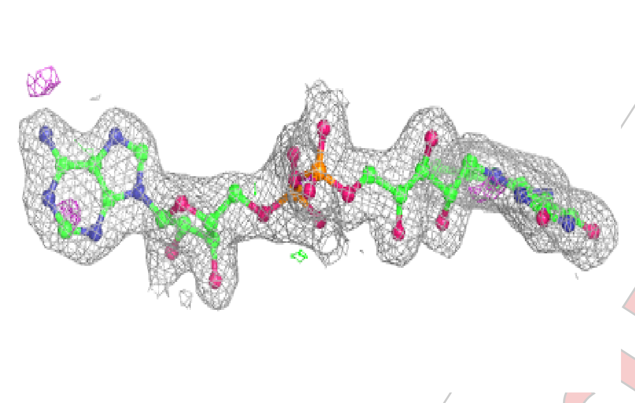

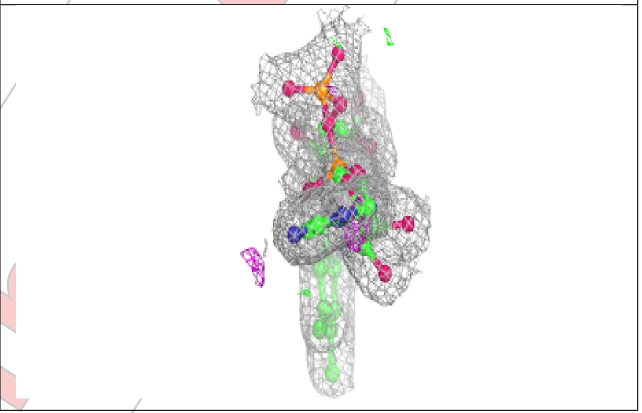

Electron density around FAD C 601:

$2mF_o\text{-}DF_c$ (at 0.7 rmsd) in gray
$mF_o\text{-}DF_c$ (at 3 rmsd) in purple (negative)
and green (positive)

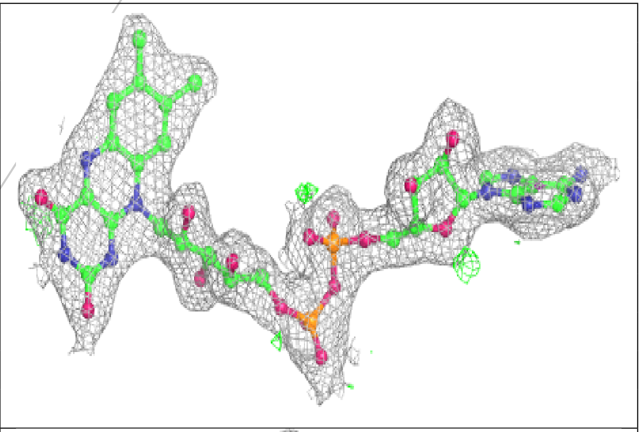

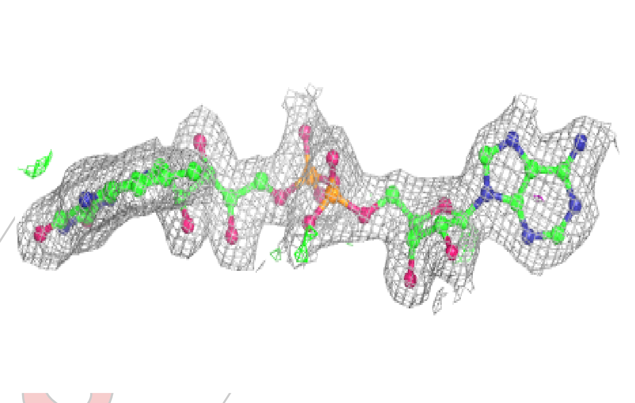

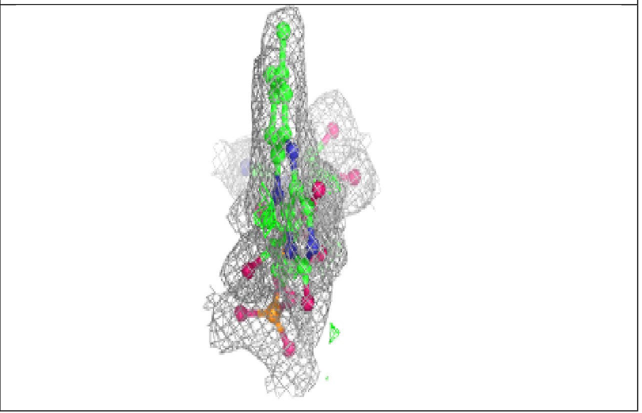

Electron density around FAD A 601:

2mF$_o$-DF$_c$ (at 0.7 rmsd) in gray
mF$_o$-DF$_c$ (at 3 rmsd) in purple (negative)
and green (positive)

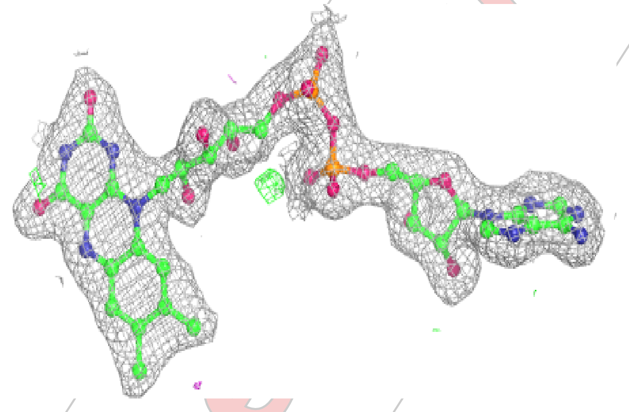

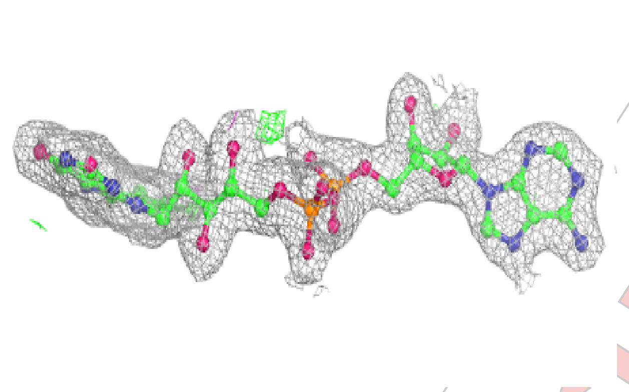

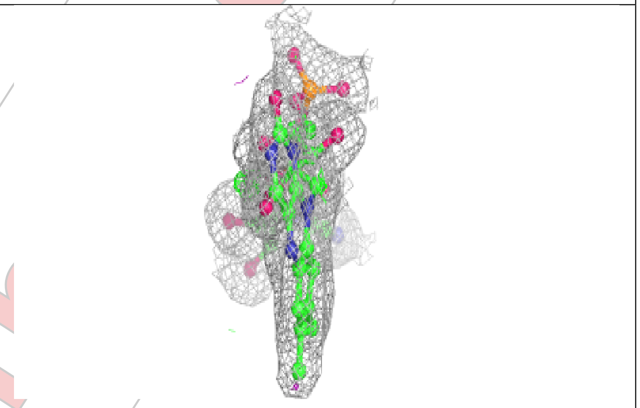

6.5 Other polymers (i)

There are no such residues in this entry.

For Manuscript Review
